# Evaluation of Two Levels of Trace Mineral Supplementation to Beef Calves Prior to Weaning

**DOI:** 10.3390/ani14192875

**Published:** 2024-10-06

**Authors:** Gracia M. P. Hernandez, Aline C. R. Dos Santos, Matheus F. L. Ferreira, David Bohnert, Juliana Ranches

**Affiliations:** 1 Eastern Oregon Agricultural Research Center, Oregon State University, Burns, OR 97720, USA; g.puertohernandez@wsu.edu (G.M.P.H.); rezendal@oregonstate.edu (A.C.R.D.S.); dave.bohnert@oregonstate.edu (D.B.); 2 Hill Farm Research Station, Louisiana State University, Homer, LA 71040, USA

**Keywords:** ceruloplasmin, cortisol, copper, haptoglobin, micro minerals, preconditioning, selenium, zinc

## Abstract

**Simple Summary:**

The trace mineral nutrition of ruminants has been widely studied; however, most of the research has focused on the requirements of mature cattle, and the requirements of trace minerals for calves prior to weaning are poorly understood. This study investigated how two levels of trace mineral supplements provided to beef calves before weaning affect their performance and mineral status. Apart from Cu, which is often a limited trace mineral in many regions, the supplementation of trace minerals above the NASEM (2016) recommendations did not improve the mineral status of beef calves.

**Abstract:**

In this 2-year study, approximately 84 days prior to weaning, 24 calves/year (Angus × Hereford) were randomly assigned to one of two treatments: trace mineral (Cu, Se, and Zn) supplementation following NASEM (2016) recommendations (Control) or trace mineral supplementation above NASEM (2016) recommendations (Super). Calves were individually fed, and trace minerals were provided in 0.5 kg of dry distiller’s grains three times weekly. Body weight (BW), blood, and liver samples were collected on d 0 and at weaning (d 84). Additional BW and blood samples were collected post-weaning on d 85, 87, 88, 91, 95, and 99 during the preconditioning phase. Initial liver concentrations of Se, Cu, and Zn were similar between treatments (*p* ≥ 0.69). At weaning, a year effect (*p* < 0.001) and a tendency for treatment × year effect (*p* = 0.09) were observed for Cu liver concentration. In year 2, but not in year 1, calves assigned to the Super treatment tended to have greater liver Cu concentration than calves assigned to the Control treatment. Except for Cu, a notoriously limited trace mineral in multiple geographical locations, the supplementation of trace minerals above the NASEM (2016) recommendations did not improve the mineral status of calves in this environment.

## 1. Introduction

Trace minerals are required in small amounts and are essential for animal growth, development, and overall health [1,2,3]. In particular, copper, selenium, and zinc (Cu, Se, and Zn, respectively) are known for their numerous roles in metabolism, including energy metabolism, enzymatic reactions, antioxidant defenses, and immune response [4,5].

In a production cycle, it is estimated that 85% of beef cattle diets derive from forages and the remaining 15% derive from byproducts consistent with feedlot diets [6]. These conventional feeds, such as forages, grains, and byproducts, occasionally fail to provide adequate concentrations of trace minerals to meet the needs of growing calves [1,7]. Research conducted by Ganskopp and Bohnert [8] reported the seasonal variation in trace mineral concentrations of native rangeland grasses and highlighted that Cu and Zn concentrations were much lower than the current requirements for beef cattle [7]. Mayland and Shewmaker [9] reported that annual and perennial grasses growing in the semiarid, west-central North American continent mature by early to mid-summer, and forage mineral concentrations in these C3 grasses decrease curvilinearly as the season advances. For tropical grasses (C4 grasses), McDowell and Arthington [10] reported that grasses growing in tropical regions can rarely completely satisfy all mineral requirements of cattle, often being deficient in a number of the macro and micro minerals. Regardless of location, when forages do not meet the mineral requirements of cattle, supplementation strategies are essential to prevent cattle mineral deficiencies and optimize performance.

Mineral requirements of cattle and supplementation strategies have been extensively studied [7]. However, most of the research focus has been towards mature cattle, with limited research conducted to understand the mineral requirements of growing calves and supplementation strategies for this particular category [11]. In a study conducted by Ranches and collaborators [11] found that beef calves consuming trace minerals (Cu, Mn, Se, and Zn) ad libitum prior to weaning failed to improve their mineral status and were considered Cu- and Se-deficient at weaning even when consuming trace minerals above the NASEM [7] recommendations for mature cattle. These data suggest a discrepancy in trace mineral requirements among cattle categories, where growing cattle might require greater amounts of trace minerals than mature cattle.

We hypothesized that the trace mineral requirements of calves prior to weaning are greater than the current NASEM [7] recommendations for mature cattle. Further, calves supplemented at NASEM [7]-recommended levels are more likely to develop mineral deficiencies at weaning, which could result in poor health and performance in the post-weaning phase. Therefore, the objective of this study was to evaluate mineral status at weaning and the subsequent post-weaning performance during the preconditioning of beef calves supplemented prior to weaning, with limit-fed supplementation fortified with trace minerals (Cu, Se, and Zn) at the NASEM [7] recommendations or above.

## 2. Materials and Methods

All animal care and handling procedures were approved by the Institutional Animal Care and Use Committee of Oregon State University (IACUC-2020-0073). This study was conducted at the Eastern Oregon Agricultural Research Center—Oregon State University (EOARC, Burns, OR; 43°51′86″ N to 119°02′15″ W) for two consecutive years, 2020 and 2021, from summer to fall (July to November).

### 2.1. Animal Management and Treatment Allocation

All study procedures were similar in both years. Approximately 84 days prior to weaning, 24 calves/year (Angus × Hereford) were randomly assigned to one of two treatments (d0), Control: trace mineral supplementation (Cu, Se, and Zn) was provided based on current guidelines of nutrient requirements for beef cattle (NASEM, 2016; 10.0, 0.10, and 30.0 mg/kg of DM, respectively, for Cu, Se, and Zn); or Super: trace mineral supplementation was provided above the current guidelines of nutrient requirements for beef cattle (NASEM, 2016; 20.0, 0.20, and 60.0 mg/kg of DM, respectively, for Cu, Se, and Zn). Trace mineral sources used for supplementation were copper sulfate, sodium selenite, and zinc sulfate, which were intended to reflect the most common commercial supplementation practices.

Trace minerals were offered three times weekly; therefore, the weekly total of trace minerals was calculated for each treatment and then offered to calves in three feeding events. Every Monday, Wednesday, and Friday, calves were brought to individual feeding pens and were individually supplemented with respective treatments. During feeding events, trace minerals were mixed with 0.5 kg of dry distiller grains (DDGs) to ensure full consumption. Calves had consistent consumption of supplement (DDG + treatment) within two weeks of supplementation. The daily average supplementation of trace minerals was calculated assuming a forage intake of 1.25% of body weight (NASEM, 2016) and was 26 and 52 mg/d of Cu, 0.26 and 0.52 mg/d of Se, and 78 and 156 mg/d of Zn, respectively, for Control and Super.

During the 84 days prior to weaning, calves were kept in two pastures (*Medicago sativa*, recently harvested for hay) with their dams and had free access to white stock salt and water. The trace mineral concentration of pastures was analyzed in a commercial laboratory (Dairy One Forage Laboratory, Ithaca, NY, USA) and was presented as averages over the two years of the study: 3.0, 0.10, and 14.0 mg/kg of DM, respectively, for Cu, Se, and Zn.

On d 84, calves were weaned, and trace mineral supplementation was ceased. Immediately after weaning, calves were moved to individual pens (≥7 × 20 m) where they had access to whole corn (0.9 kg per calf, daily) and ad libitum access to chopped mixed alfalfa-grass hay for 21 days in a preconditioning program. All pens had concrete feed bunks (7 m) and automatic water tanks (Brower^®^, 71 × 35.5 × 66 cm).

### 2.2. Sample Collections

Body weights of calves were measured at the beginning of the study (d 0), at weaning (d 84 and 85), and at the end (a final BW averaged over d 105 and 106). Full BW was collected to avoid shrink-induced stress, and, therefore, for weaning (d 84 and 85) and final body weight (d 105 and 106), the average BW measured on two consecutive days was used. 

Liver tissue samples for the evaluation of trace mineral concentrations were collected on d 0 and d 84 by a trained technician using techniques previously described [12]. Samples were collected between the 10th and 11th intercostal space using a Tru-Cut biopsy needle (CareFusion, 14-gauge × 15 cm; Becton Dickinson, Vernon Hills, IL, USA). Four core tissue samples were collected from each animal. Following collection, samples were frozen at −20 °C and sent to an analytical laboratory for mineral analyses (Michigan State University, Animal Health Diagnostic Laboratory, Lansing, MI, USA).

Blood samples were collected at weaning (d 84) and on d 85, 87, 88, 91, 95, and 99 via jugular vein using commercial heparinized vacuum tubes for plasma harvest (BD Vacutainer, 10 mL; Becton, Dickinson and Company, Franklin Lakes, NJ, USA). Blood samples were placed on ice immediately following collection and centrifuged at 2500× *g* for 30 min at 4 °C for plasma harvest. Samples were frozen at −20 °C and stored at −80 °C until further analysis.

During the preconditioning phase, hay samples were collected weekly, and composite samples were made for the entire post-weaning period. Samples were dried in a forced-air oven (55 °C) for 72 h, ground at 1 and 2 mm in a Wiley mill (model 3, Arthur H. Thomas, Philadelphia, PA, USA), and sent to a commercial laboratory (Dairy One Forage Laboratory, Ithaca, NY) for nutrient analyses. Samples were analyzed by wet chemistry for crude protein (CP; method 984.13; [13], neutral digestible fiber (NDF; [14]) modified for use in an Ankom 200 fiber analyzer (Ankom Technology Corp., Macedon, NY, USA), and total digestible nutrients (TDNs; [15]). Alfalfa-grass hay samples were reported as 14.5% for CP, 47.9% for NDF, and 67% for TDNs.

### 2.3. Laboratory Analysis

Blood samples collected during the preconditioning phase were evaluated for cortisol, ceruloplasmin, and haptoglobin concentrations as markers of stress and acute-phase response [16].

Plasma cortisol concentrations were measured in a single run using chemiluminescent enzyme immuno-assays (Immulite 1000; Siemens Medical Solutions Diagnostics, Los Angeles, CA, USA) with an intra-assay CV of 9.04%.

Plasma ceruloplasmin oxidase activity was measured in duplicate samples using colorimetric procedures previously described [17]. Ceruloplasmin concentrations were expressed as mg/dL as described [18]. The intra- and inter-assay CVs were 3.17 and 7.64%, respectively. 

Plasma haptoglobin concentrations were determined in duplicate samples by a biochemical assay measuring the haptoglobin–hemoglobin complex by estimating differences in peroxidase activity [19]. Results were obtained as arbitrary units resulting from reading plates at 450 nm (VersaMax^®^ Turnable EXT). The same quality control standards used in the biochemical assay were analyzed by the quantitative determination of bovine haptoglobin in plasma (bovine haptoglobin ELISA test kit; Life Diagnostics, Inc., West Chester, PA, USA). The concentration of haptoglobin, based on the ELISA assay, ranged from 0.03 (low control) to 0.95 mg/mL (high control), with an intra-assay coefficient of variability (CV) of 1.26%. The ELISA standard curve was used to convert the arbitrary units obtained from the biochemical procedures into mg/mL with the least detectable value of 0.03 mg/mL [20]. The intra- and inter-assay CVs were 2.28 and 7.46%, respectively.

### 2.4. Statistical Analysis

All data were analyzed using the MIXED procedure of SAS (SAS Inst. Inc., Cary, NC, USA; Version 9.4). For all variables, a calf was considered the experimental unit. The model statement included treatment, year, and possible interactions. Blood, liver, and BW measurements were analyzed as repeated measures, where the calf was the experimental unit, and the model statement included treatment, day, and year. Initial body weight was used as a covariate for all body weight variables. Compound symmetry covariance structure was used for the repeated measures analyses, as this covariance structure generated the lowest Akaike information criterion. Data were separated using PDIFF if a significant preliminary F-test was detected. Significance was set at *p* ≤ 0.05, and tendencies if *p* > 0.05 and ≤0.10.

## 3. Results

No treatment differences (*p* ≥ 0.11) were observed for initial BW or weaning weight, BW at d 15 post-weaning, final BW, or for ADG pre- or post-weaning (Table 1).

As per the study design, no treatment differences (*p* ≥ 0.69) were observed for the initial liver concentrations of Cu, Se, and Zn. Calves assigned to both treatments successfully maintained adequate mineral status until weaning (40.0–650.0 mg/kg, 0.60–3.30 mg/kg of DM, 90.0–500.0 mg/kg, respectively, for Cu, Se, and Zn; VDL MSU reference ranges), regardless of treatment.

At weaning, no treatment differences were observed for liver Se (*p* = 0.54) or Zn (*p* = 0.18) concentration. A year effect (*p* < 0.001) and a tendency for treatment × year effect (*p* = 0.09) was observed for liver Cu concentration at weaning. In year 2 but not in year 1, calves assigned to the Super treatment tended to have greater liver Cu concentration than calves assigned to the Control treatment (Table 2).

**Figure 1:**
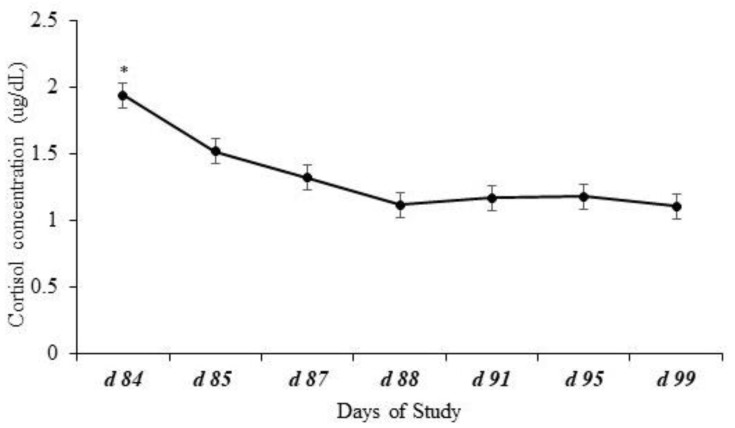
Plasma cortisol concentration of calves at weaning (d 84) and post-weaning. Effects of day represented by * (*p* < 0.0001) but no effects of treatment (*p* = 0.31), year (*p* = 0.40), or the possible interactions (*p* ≥ 0.78) were observed for plasma cortisol concentration. Plasma cortisol concentration peaked (*p* ≤ 0.04) at weaning (d 84) and decreased over time until the last day of blood collection.

Effects of day (*p* < 0.0001) but no effects of treatment (*p* = 0.31), year (*p* = 0.40), or interactions (*p* ≥ 0.78) were observed for plasma cortisol concentration. Plasma cortisol concentration peaked (*p* ≤ 0.04) at weaning (d 84) and decreased consistently until the last collection on d 99 (Figure 1). 

**Figure 2:**
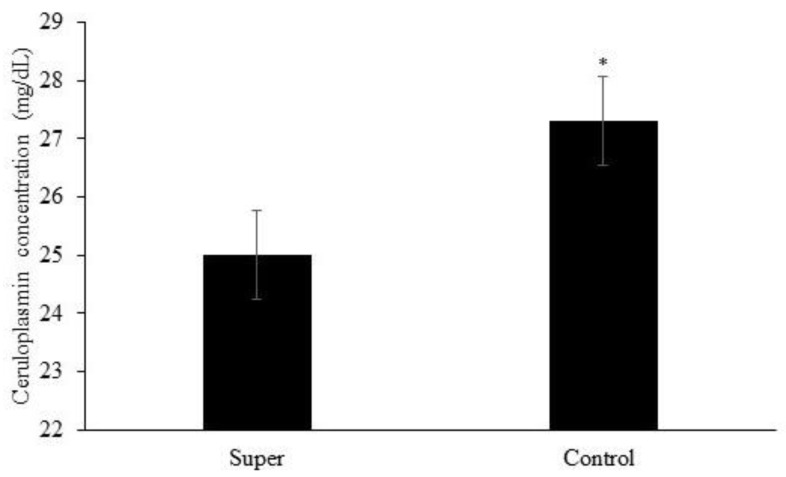
Mean plasma concentration of ceruloplasmin of calves post-weaning. Effects of treatment (*p* = 0.04) represented by * and year (*p* = 0.0003) but no effects of treatment × year (*p* = 0.54) or the other possible interactions (*p* ≥ 0.15) were observed for plasma ceruloplasmin concentration.

Effects of treatment (*p* = 0.04) and year (*p* = 0.0003) but no effects of treatment × year (*p* = 0.54) or the other possible interactions (*p* ≥ 0.15) were observed for plasma ceruloplasmin concentration. Plasma ceruloplasmin concentration was greater for calves assigned to the Control treatment when compared to calves assigned to the Super treatment (Figure 2). Plasma ceruloplasmin concentration was greater in year 1 vs. year 2 (24.0 vs. 28.2 mg/dL, respectively; not shown). 

**Figure 3:**
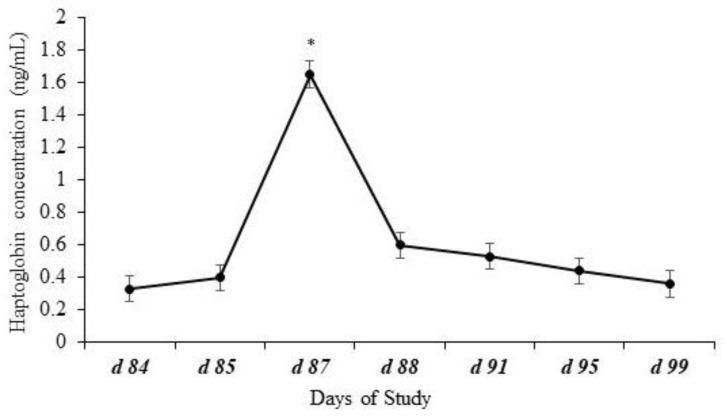
Plasma haptoglobin concentration of calves at weaning (d 84) and post-weaning. Effects of day (*p* < 0.0001) represented by * but no effects of treatment (*p* = 0.98), year (*p* = 0.18), or the possible interactions (*p* ≥ 0.69) were observed for plasma haptoglobin concentrations. Plasma haptoglobin concentration showed a common pattern, peaking after weaning.

Effects of day (*p* < 0.0001) but no effects of treatment (*p* = 0.98), year (*p* = 0.18), or other possible interactions (*p* ≥ 0.69) were observed for plasma haptoglobin concentrations. Plasma haptoglobin concentration had an expected pattern, peaking after weaning on d 87 (Figure 3).

## 4. Discussion

Trace mineral supplementation of cattle is often associated with improved performance, immunity, and reproduction. However, improvement of those traits is most often observed when cattle are considered mineral deficient or when nutritional strategies are implemented during challenging periods [2,3,4,10].

In the current study, calf growth was not changed by the level of trace mineral supplementation, regardless of the level of supplementation. This lack of treatment effect on animal performance is not surprising and has been observed by others [11,21,22,23]. Stanton et al. [23] reported improved weight gain for calves supplemented above current recommendations when providing organic sources of trace minerals. Differently from the current study, in that study, calves had free-choice access to mineral supplements. Additionally, dams of the respective calves were supplemented with organic trace minerals during late gestation, which could have led to a fetal programming effect and therefore resulted in the improved weight gain of calves, as observed by others [24]. Research evaluating the supplementation of trace minerals to calves prior to weaning is limited; however, Ranches et al. [11] reported that calves consuming trace minerals above NASEM [7] recommendations prior to weaning for approximately 90 days were considered Cu- and Se-deficient at weaning (40.0–650.0 mg/kg, 0.60–3.30 mg/kg of DM, 90.0–500.0 mg/kg, respectively, for Cu, Se, and Zn; VDL MSU reference ranges). In disagreement, this study found that regardless of treatment, calves maintained adequate trace mineral status from the beginning of supplementation until weaning. Although these studies have similarities, such as the length of supplementation and the age of the calves, the studies also have large discrepancies, such as the breed of calves, pasture quality and quantity, and the presence of antagonists in forages and feed, all factors well known to affect the mineral status of cattle [2,10]. Most importantly, the levels of supplementation used in this study were very conservative, especially when compared to the calculated intake observed previously [11], where calves were consuming approximately 122 and 5.5 mg/d of Cu and Se, emphasizing the effects of environmental factors on mineral status.

Supporting this study’s hypothesis, calves assigned to the Super treatment tended to have greater liver Cu concentration at weaning in year 2 when compared to calves assigned to the Control treatment. Similarly, a dose response for heifers supplemented with different sources of Cu. In that study, heifers supplemented with 30 mg/kg of Cu had greater Cu liver concentration than heifers supplemented with 15 mg/kg of Cu was reported [25]. In another study, Engle and Spears [26] also demonstrated a dose response for Cu supplementation, in which growing steers supplemented with 40 mg/kg of Cu had greater Cu liver concentration than steers supplemented with 20 mg/kg of Cu. Nonetheless, Stoszek et al. [27] reported that dietary Cu supplementation was positively correlated with the accumulation of liver Cu; however, the rate of increase in liver Cu concentration diminished at higher supplementation rates, suggesting an efficient mechanism preventing fast, toxic Cu overloading in cattle.

Interestingly, the plasma ceruloplasmin concentration of calves assigned to the Control treatment was greater than that of calves assigned to the Super treatment. Plasma ceruloplasmin holds the majority of Cu found in plasma (90% to 95%; Cousins, 1985), making it an important indicator of Cu status. Additionally, plasma ceruloplasmin concentration has been reported to be responsive to Cu supplementation or the lack thereof [28]. Nevertheless, ceruloplasmin is an acute-phase protein, which is responsive to an array of insults, such as inflammation, bacterial infection, and physical injury [16]. Therefore, it is reasonable that calves assigned to the Control treatment, having lower Cu liver concentration, experienced a more pronounced immune response at weaning, resulting in a greater concentration of circulating ceruloplasmin, while calves assigned to the Super treatment had improved Cu status and, therefore, less circulating ceruloplasmin. Interestingly, López-Alonso et al. [29] reported that for cattle with moderate liver Cu accumulation, neither the serum Cu nor the ceruloplasmin concentration was correlated to Cu liver concentration. Ranches et al. [30] also found that, regardless of the liver Cu concentration (deficiency vs. adequacy), only whole-blood Cu concentration correlated to Cu liver concentration. Finally, Claypool et al. [31] also reported the lack of concordance between blood and liver Cu. 

Stress at weaning has been extensively studied [32,33], and often, blood markers, such as cortisol, and acute-phase proteins, such as ceruloplasmin and haptoglobin, are used as a welfare assessment tool [16,34]. In the present study, plasma cortisol, ceruloplasmin, and haptoglobin concentrations had a typical response as observed in other studies, in which cortisol concentrations increase immediately after weaning, while the acute-phase proteins increase 48 to 72 h after weaning [11,22,35,36,37]. Although no treatment effects were observed for cortisol or haptoglobin concentration, the greater concentration in plasma ceruloplasmin observed for calves assigned to the Control treatment suggests that weaning was more challenging to calves assigned to the Control treatment, resulting in greater ceruloplasmin concentration and lesser Cu liver concentration. 

## 5. Conclusions

In summary, no changes in performance were observed when supplementing different levels of trace minerals to pre-weaned calves. With the exception of Cu, it does not seem that the supplementation of trace minerals above the current NASEM (2016) recommendations leads to greater mineral status for calves in the current environmental conditions. However, greater trace mineral supplementation seems to ameliorate the concentration of blood markers associated with weaning stress.

## Figures and Tables

**Table 1:** Body weight and average daily gain of beef calves supplemented at two different levels of trace minerals (Cu, Se, and Zn) prior to weaning for 84 days.

	Treatments ^1^			
Item	Control	Super	SEM	*p*-Value
Initial BW, kg ^2^	175	169	6.17	0.17
Weaning BW, kg ^2^	276	268	3.52	0.12
Final BW, kg ^2^	283	275	3.69	0.11
Pre-weaning ADG, kg ^3^	1.19	1.16	0.093	0.73
Post-weaning ADG, kg ^4^	0.414	0.348	0.073	0.53

^1^ Approximately 84 days prior to weaning, 24 calves/year (Angus × Hereford) were randomly assigned to one of two treatments (d 0), Control: trace mineral supplementation (Cu, Se, and Zn) was provided based on current guidelines of nutrient requirements for beef cattle (NASEM, 2016; 10.0, 0.10, and 30.0 mg/kg of DM, respectively, for Cu, Se, and Zn); or Super: trace mineral supplementation was provided above the current guidelines of nutrient requirements for beef cattle (NASEM, 2016; 20.0, 0.20, and 60.0 mg/kg of DM, respectively, for Cu, Se, and Zn). ^2^ Initial, weaning, and final body weights are full body weights measured on two consecutive days. Therefore, data are presented as an average of both weights. Initial body weight was used as a covariate for all body weight variables. ^3^ Pre-weaning average daily gain was calculated using weaning body weight minus initial body weight divided by 84 days. ^4^ Post-weaning average daily gain was calculated for the post-weaning phase, using final body weight minus weaning body weight and then divided by 21 days.

**Table 2:** Mineral status of beef calves supplemented at two different levels of trace minerals (Cu, Se, and Zn) prior to weaning for 84 days.

		Treatments ^1^			
Item	Year ^4^	Control	Super	SEM	*p*-Value
Initial Cu, mg/kg ^2^		120	122	12.2	0.90
Initial Se, mg/kg ^2^		1.09	1.13	0.086	0.69
Initial Zn, mg/kg ^2^		209	213	17.3	0.81
Weaning Cu, mg/kg ^3^	Year 1	67	53	16.4	0.52
	Year 2	127	167	16.4	0.08
Weaning Se, mg/kg ^3^		1.07	1.00	0.077	0.54
Weaning Zn, mg/kg ^3^		158	138	10.6	0.18

^1^ Approximately 84 days prior to weaning, 24 calves/year (Angus × Hereford) were randomly assigned to one of two treatments (d0), Control: trace mineral supplementation (Cu, Se, and Zn) was provided based on current guidelines of nutrient requirements for beef cattle (NASEM, 2016; 10.0, 0.10, and 30.0 mg/kg of DM, respectively, for Cu, Se, and Zn); or Super: trace mineral supplementation was provided above the current guidelines of nutrient requirements for beef cattle (NASEM, 2016; 20.0, 0.20, and 60.0 mg/kg of DM, respectively, for Cu, Se, and Zn). ^2^ Initial liver samples were collected on d0 of the study. Samples were sent to an analytical laboratory for mineral analyses (Michigan State University, Animal Health Diagnostic Laboratory, Lansing, MI, USA; Reference range: Cu: 40–650 mg/kg; Se: 0.60–3.30 mg/kg; Zn: 90–500 mg/kg). ^3^ A second liver sample was collected at weaning to evaluate the effects of trace mineral supplementation through the mineral status of calves. ^4^ Effects of year (*p* < 0.001) and treatment × year (*p* = 0.09) were observed for Cu concentration at weaning.

## Data Availability

The data presented in this study are available upon request from the corresponding author.
